# Metabolic challenges in rheumatoid arthritis: a translational overview from pathogenesis to patient care

**DOI:** 10.1007/s00592-026-02694-5

**Published:** 2026-04-15

**Authors:** Sara Ferrigno, Eneida Çela, Mauro Fatica, Benedetta Monosi, Arianna D’Antonio, Paola Conigliaro, Marina Cardellini, Susanna Longo, Massimo Federici, Maria Sole Chimenti

**Affiliations:** 1 Rheumatology, Allergology and Clinical Immunology, Department of Systems Medicine, Department of “Medicina dei Sistemi”, University of Rome Tor Vergata, Via Montpellier 1, Rome, 00133 Italy; 2 Academic Rheumatology Unit, Department of Medicine and Health Sciences “Vincenzo Tiberio”, University of Molise, Via Giovanni Paolo II, C/da Tappino, Campobasso, 86100 Italy; 3 Department of Systems Medicine, University of Rome Tor Vergata, Via Montpellier 1, Rome, 00133 Italy; 4 Center for Atherosclerosis, Policlinico Tor Vergata, Viale Oxford 81, Rome, 00133 Italy

**Keywords:** Rheumatoid arthritis, Glucose metabolism, Lipid metabolism, Atherogenesis, Inflammation, Immune-metabolism, Cardiovascular risk

## Abstract

Rheumatoid arthritis (RA) is an inflammatory disease characterized by a higher burden of cardiovascular and metabolic diseases than in the general population. Altered lipid and glucose metabolic pathways are widely observed, primarily due to chronic inflammation. However, metabolic dysfunction may also affect RA pathogenesis, further enhancing immune cell activation and joint damage. Glucose and lipid alterations observed in RA help define the comorbidity burden of this disease, significantly affecting disease activity and prognosis. The aim of the present review is to describe the role of metabolic dysfunctions in RA and to examine how disease activity and treatments can influence these conditions. We also summarized the main management strategies based on current literature and developed a cardiometabolic monitoring algorithm across different clinical settings to support daily patient care of these patients.

## Introduction

Rheumatoid arthritis (RA) is a chronic immune-mediated inflammatory disease associated with a significantly increased metabolic and cardiovascular (CV) risk [1].

Chronic systemic inflammation affects metabolic homeostasis by impairing insulin sensitivity and glucose and lipoprotein metabolism. Inflammation may influence and remodel conventional metabolic markers, leading to a paradoxical lipid profile and to potential underestimation of CV risk. Moreover, metabolic abnormalities can interact with immune and inflammatory pathways, contributing to disease-related mechanisms [2]. This two-way relationship between inflammation and metabolism has led to the concept of “immunometabolism”, in which metabolic pathways actively modulate immune responses.

Recent integrative analyses have raised attention toward a new approach to cardiometabolic comorbidities in RA [3]. This narrative review provides an updated synthesis of mechanisms linking inflammation to metabolic dysfunction and discusses their impact on clinical practice.

Pharmacological treatment further complicates this interplay. Anti-rheumatic therapies, including glucocorticoids and disease-modifying anti-rheumatic drugs (DMARDs), exert heterogeneous effects on glucose and lipid metabolism, with a potential higher risk of type 2 Diabetes Mellitus (T2DM) and CV diseases. Recently, attention has also been raised toward the metabolic and potential immunomodulatory effects of new glucose-lowering agents in chronic inflammatory conditions [4].

In this narrative review, we describe cardiometabolic changes in RA arising from three main directions: inflammation-driven mechanisms related to the underlying disease pathogenesis, patient-related cardiometabolic risk factors, and metabolic effects of antirheumatic therapies.

We also integrate current evidence on immune-driven metabolic alterations in glucose and lipid metabolism in RA and their clinical effects on cardiometabolic risk assessment and management. Finally, we propose a practical cardiometabolic monitoring algorithm to support clinicians involved in the multidisciplinary care of patients with RA.

## Methods

This narrative literature review was developed through a targeted, non-systematic search of PubMed/MEDLINE, focusing on articles published from 2000 to 2025. Search terms included: rheumatoid arthritis, insulin resistance, glucose metabolism, glycaemic control, dyslipidaemia, lipid profile, cardiovascular risk, inflammation, glucocorticoids, conventional DMARDs, biologic DMARDs, targeted synthetic DMARDs, GLP-1 receptor agonists, and SGLT2 inhibitors. Relevant publications were identified through accurate screening of the reference lists.

Systematic review, meta-analyses, randomized controlled trials, large observational cohort studies, and position statements or guidelines from major scientific societies were prioritized. Mechanistic and preclinical studies were included to support biological plausibility and to contextualize clinical observations, particularly where high-level clinical evidence was limited.

The selection of studies was guided by the relevance of metabolic outcomes, including insulin resistance (IR), glucose and lipid homeostasis, and CV risk. Another main purpose was to understand the interaction between chronic inflammation, antirheumatic therapies, and metabolic regulation. In line with the review’s narrative design, no systematic selection process or quantitative quality assessment was applied.

## Interplay between inflammatory and glucose dysregulation in RA

### Mechanisms of inflammation-induced glucose changes in RA

Numerous studies have demonstrated a correlation among IR, T2DM, and the molecular mechanisms driving RA. Pro-inflammatory cytokines, such as Tumor Necrosis Factor-alpha (TNF-α), Interleukin-6 (IL-6), and Interleukin-1 (IL-1), are central mediators of this interaction [5], by acting through an interconnected signalling network and amplifying each other’s effects on glucose metabolism.

TNF-α plays a central role in the development of IR by directly interfering with insulin receptor signalling and glucose uptake. First, it induces post-translational modifications of the insulin receptor and insulin receptor substrate-1 (IRS-1), functionally inhibiting the insulin signalling [6]. This altered phosphorylation pattern impairs activation of phosphoinositide 3-kinase (PI3K) and protein kinase B (Akt), reducing the translocation of glucose transporter type 4 (GLUT4) to the cell membrane in adipocytes and skeletal muscle cells [7] and thereby impairing glucose uptake. In parallel, TNF-α upregulates Suppressor of Cytokine Signalling 3 (SOCS-3), a known inhibitor of the insulin signalling pathway. Moreover, TNF-α inhibits the synthesis of peroxisome proliferator-activated receptor gamma (PPARγ), compromising adipocyte differentiation and insulin sensitivity. It also stimulates lipolysis and increases circulating free fatty acids (FA), thereby exacerbating IR [5].

The impact of IL-6 on glucose metabolism is variable: acute IL-6 elevation may enhance insulin sensitivity; however, chronic exposure and overproduction observed in RA promote IR through SOCS-3 upregulation and altered IRS-1 phosphorylation [8].

IL-1β exerts a similarly dual effect on glucose metabolism: at low concentrations, it enhances insulin secretion, whereas chronic exposure induces β-cell dysfunction and apoptosis via activation of the Nuclear factor kappa-light-chain-enhancer of activated B cells (NF-κB), mitogen-activated protein kinase (MAPK), and c-Jun N-terminal kinase (JNK) signalling pathways, with subsequent nitric oxide and reactive oxygen species production and higher oxidative stress. Moreover, hyperglycaemia itself amplifies IL-1β expression and inhibits IL-1 receptor antagonist production in pancreatic islets [9].

**Fig. 1:**
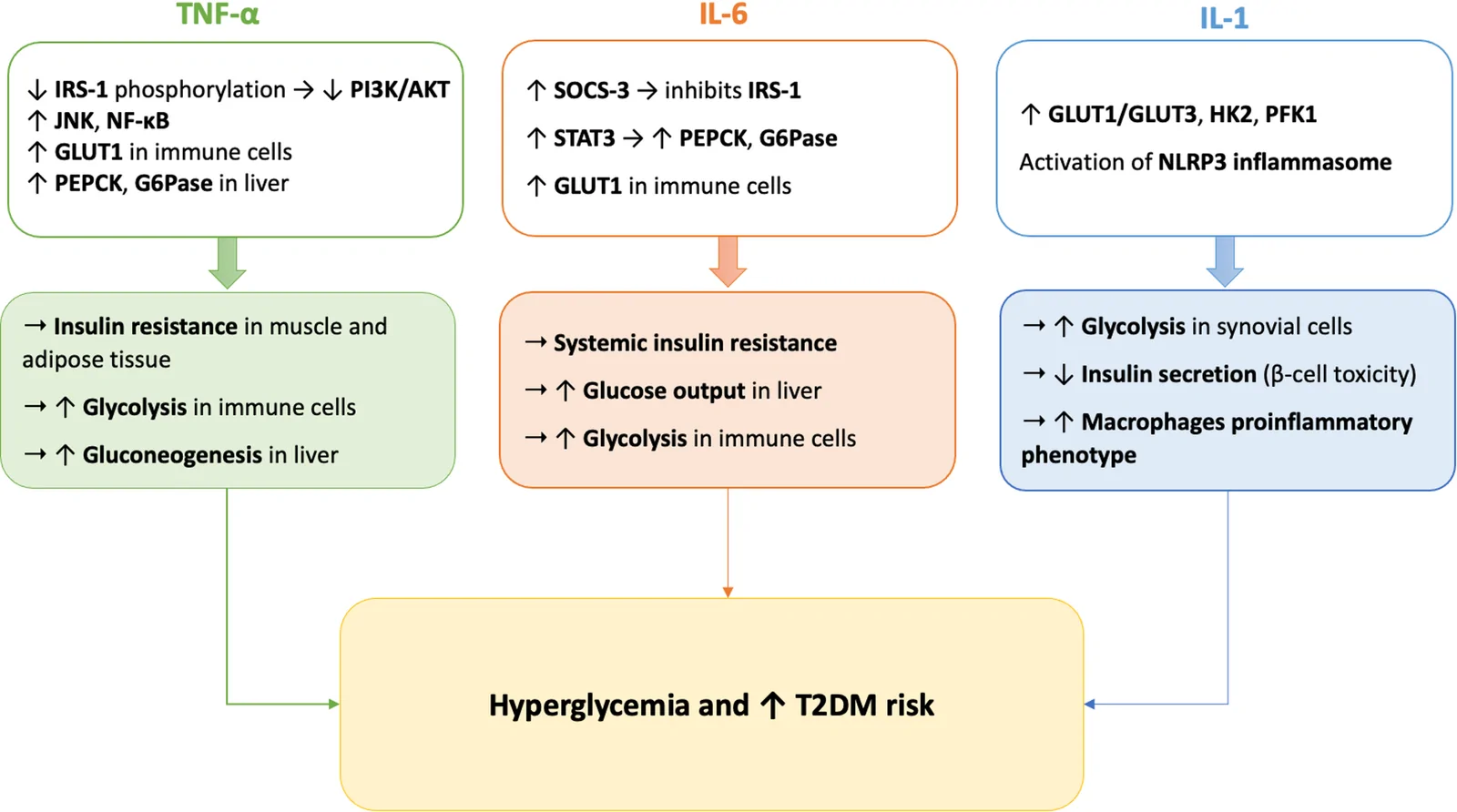
Inflammation-driven mechanisms underlying glucose metabolism alterations in Rheumatoid Arthritis (RA). Chronic inflammation in RA disrupts glucose homeostasis through the action of pro-inflammatory cytokines such as TNF-α, IL-6, and IL-1β. These mediators promote insulin resistance in peripheral tissues, impair insulin signalling and increase hepatic glucose production, while enhancing glycolytic activity in immune and stromal cells within inflamed tissues. In addition, inflammatory pathways negatively affect pancreatic β-cell function. Together, these mechanisms contribute to hyperglycaemia and an increased risk of type 2 diabetes mellitus in RA. *RA* rheumatoid arthritis, *TNF-α* tumor necrosis factor alpha, *IL-6* interleukin-6, *IL-1β* interleukin-1 beta, *IRS-1* insulin receptor substrate 1, *JNK* c-Jun N-terminal kinase, *SOCS-3* suppressor of cytokine signaling 3, *GLUT1* glucose transporter type 1, *GLUT3* glucose transporter type 3, *PEPCK* phosphoenolpyruvate carboxykinase, *G6Pase* glucose-6-phosphatase, *NLRP3* NOD-, LRR- and pyrin domain-containing protein 3, *T2DM* type 2 diabetes mellitus

Finally, activation of the Janus kinase/signal transducer and activator of transcription (JAK/STAT) pathway, which has a key role in RA pathogenesis, has been linked to upregulation of pro-apoptotic mediators and progressive loss of β-cells [10] (Fig. 1).

### Glucose metabolism in RA pathogenesis

The relationship between glucose metabolism and RA is not unidirectional; rather, hyperglycaemia and T2DM may act as catalysts that accelerate RA progression, enhancing systemic inflammation, exacerbating structural joint damage, and significantly impacting therapeutic efficacy. Metabolic Syndrome (MetS), particularly obesity, alters the pharmacokinetics of various medications, sustaining a chronic inflammatory state and it has been associated with reduced therapeutic response to TNF inhibitors in several studies [11].

At the molecular level, chronic hyperglycaemia facilitates the accumulation of advanced glycation end products (AGEs). These compounds function as pro-inflammatory ligands that activate specific immune receptors, amplifying TNF-α and IL-6 production. Within the joint microenvironment, AGEs enhance synovial inflammation and activate degradative pathways, accelerating cartilage consumption and bone erosion [12].

This structural decline is also associated with increased oxidative stress, which favours osteoclast differentiation and hyperactivity, resulting in more aggressive radiographic progression [13].

IR further promotes systemic inflammation, reducing the clinical response to both conventional synthetic (cs)DMARDs and biologics. Consequently, patients with IR often exhibit higher disease activity scores, poorer clinical outcomes, and increased rates of long-term disability [14].

These findings are also confirmed by the Cardiovascular, Obesity and Rheumatic Diseases Italian Study (CORDIS) group, which highlighted that RA patients with diabetes suffer from significantly higher functional disability. Furthermore, these individuals are more likely to require escalation to biologic (b)DMARDs compared to the others, suggesting that metabolic dysfunction resembles a more refractory disease phenotype [15] .

## Impact of RA therapies on glucose metabolism

Glucocorticoids (GC) stimulate hepatic glucogenesis, reduce insulin secretion, and induce IR. Prolonged treatment with moderate-to-high doses increases T2DM risk, while short-term high-dose therapy carries lower diabetogenic effects, with predominance of the beneficial anti-inflammatory benefits [16].

Methotrexate (MTX), a first-line csDMARD in RA, is linked to lower MetS prevalence and improved glucose control. In a study of 400 RA patients, MTX use was associated with reduced MetS and fasting blood glucose (FBG) [17]. Other csDMARDs, such as leflunomide and sulfasalazine, do not significantly affect glucose metabolism.

Hydroxychloroquine (HCQ), an antimalarial drug commonly used as a csDMARD in RA, improves insulin sensitivity and reduces the risk of T2DM [18].

Treatment with TNF-α inhibitor has been associated with a reduced IR and T2DM incidence, particularly among normal-weight patients [19]. Additionally, a recent meta-analysis reported a significant increase in Insulin Sensitivity, measured by the Quantitative Insulin Sensitivity Check Index (QUICKI), in patients treated with different anti-TNF-α regimens. According to the results, the QUICKI increased with minimal variability across the three drugs, suggesting comparable clinical efficacy [20]. Finally, the CORRONA study, a large multicentre cohort, evaluated the development of T2DM in RA patients. After adjustment for BMI, disease activity, and GC therapy, TNF-α inhibitors were associated with reduced IR and a reduced risk of T2DM [21].

Among other bDMARDs considered for RA treatment, anakinra, an IL-1 inhibitor, improved HbA1c, proinsulin-to-insulin ratio, β-cell function, and inflammatory markers in patients with T2DM. On the other side, tocilizumab, an IL-6 receptor antagonist, was associated with reduced HbA1c in RA patients, with no hypoglycaemia events. Given that serum IL-6 levels rise before IR and T2DM onset, targeting this pathway could also be promising in improving patients’ metabolic profile [22].

Finally, JAK inhibitors are highly effective in managing synovial inflammation, and clinical data showed minimal impact on glucose metabolism [23].

## Interplay between inflammatory and lipid dysregulation in RA

### Mechanisms of inflammatory-induced lipid changes

Dyslipidaemia is observed in approximately 55–65% of RA patients and may already be detectable during the preclinical phase of the disease, preceding overt joint involvement. RA patients carry an increased CV risk, mainly due to accelerated atherosclerosis. However, lipid abnormalities in RA differ substantially from those observed in the general population and are strongly related to the chronic inflammation characterizing the disease. During high disease activity, RA patients frequently show reduced levels of total cholesterol and low-density lipoprotein cholesterol (LDL), a phenomenon commonly referred to as the “lipid paradox”, since these lower lipid concentrations are paradoxically associated with increased CV risk [24]. This pattern is secondary to alterations in lipid turnover induced by inflammatory cytokines, such as TNF-α, IL-6 and IL-1β, rather than a truly protective lipid profile [25].

Beyond quantitative changes, chronic inflammation alters lipoprotein composition and function. Inflammatory microenvironment impairs high-density lipoprotein (HDL) maturation by inhibiting lecithin–cholesterol acyltransferase (LCAT) activity. Particularly, HDL shifts toward a dysfunctional, pro-inflammatory phenotype characterized by reduced cholesterol efflux capacity and loss of antioxidant properties [26]. Apolipoprotein A-I (ApoA-I), which constitutes the major protein component of HDL and plays a central role in reverse cholesterol transport, is reduced in RA due to decreased hepatic synthesis and increased degradation [27]. In addition, oxidative modifications of ApoA-I driven by elevated myeloperoxidase (MPO) activity impair its interaction with ATP-binding cassette transporter A1 (ABCA1), further limiting cholesterol efflux [28].

**Fig. 2:**
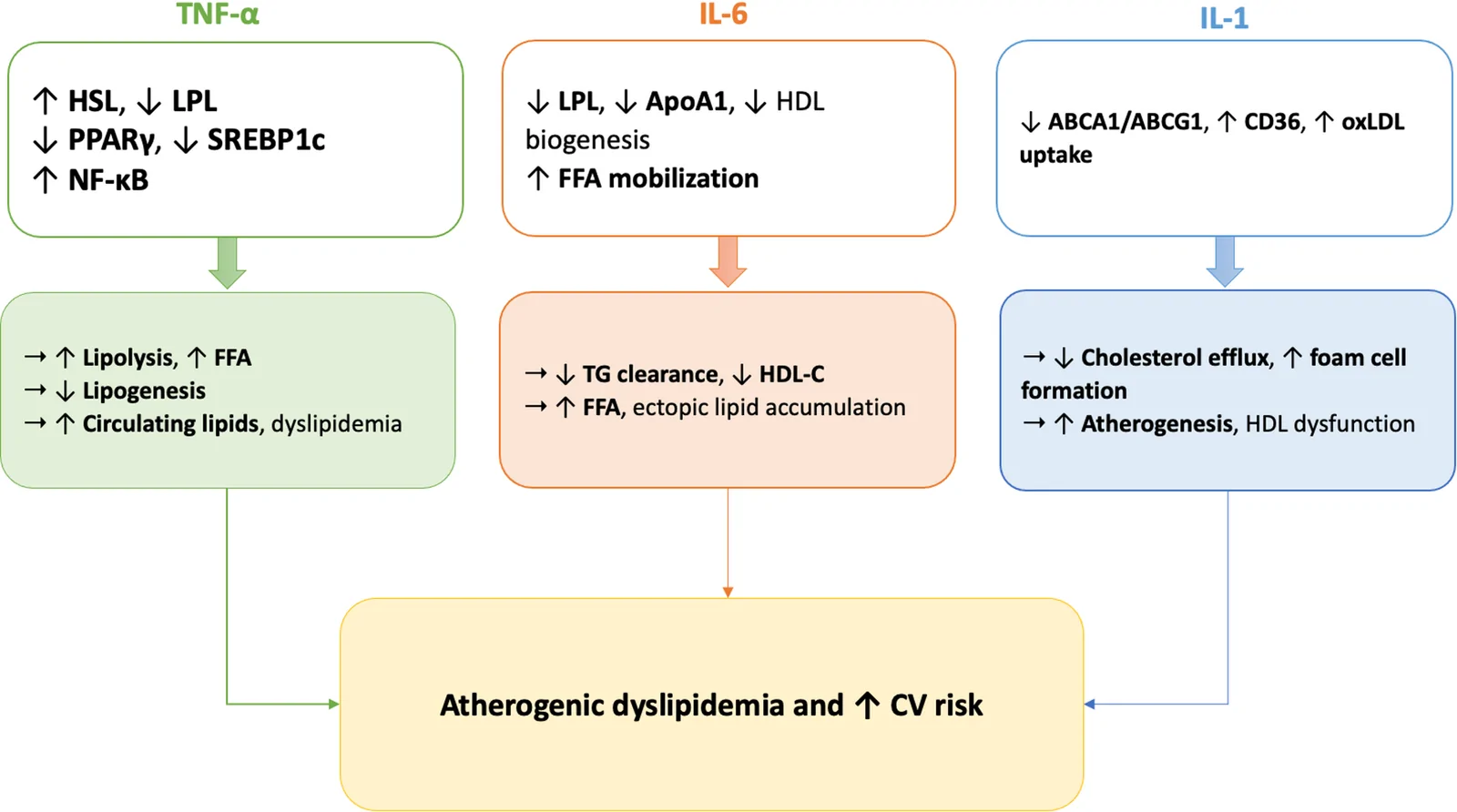
Inflammation-driven mechanisms underlying lipid metabolism alterations in Rheumatoid Arthritis. Chronic inflammation in rheumatoid arthritis alters lipid metabolism and promotes a pro-atherogenic profile. Cytokines as TNF-α, IL-6 and IL-1β enhance lipid mobilization, impair lipoprotein clearance and contribute to qualitative alterations in high-density lipoproteins. They also amplify oxidative stress, resulting in higher LDL oxidation, higher uptake of lipids from macrophages, leading to atherosclerotic plaque formation. *HSL* hormone-sensitive lipase (HSL), *FFA* free fatty acids, *SREBP-1c* sterol regulatory element-binding protein-1c, *LPL* lipoprotein lipase, *PPARγ* Peroxisome Proliferator-Activated Receptor Gamma, *NF-kB* nuclear factor kappa-light-chain-enhancer of activated B cells, *ApoA1* Apolipoprotein A1, *HDL-C* High-Density Lipoprotein Cholesterol, *TG* triglycerides, *ABCA1/ABCG1* ATP-Binding Cassette Transporters A1/G1, *LDL* Low-Density Lipoprotein, *CV* cardiovascular

Oxidative stress represents a key amplifier of lipid alterations in RA. Increased production of reactive oxygen species and MPO promotes LDL oxidation in both serum and synovial fluid, favouring macrophage uptake and foam cell formation [29]. In parallel, inflammatory cytokines modulate hepatic lipid handling, enhancing LDL clearance from the circulation and facilitating lipid deposition within the arterial wall [30]. Role of triglycerides (TG) has not been completely elucidated; however, their levels appear to be elevated in early active disease and reduced in the chronic phases [31] (Fig. 2).

### Lipid metabolism in RA pathogenesis

Current evidence in RA suggests that lipid metabolic alterations may also actively contribute to disease pathogenesis. Lipidomic analyses identified a distinct metabolic signature in RA, characterized by an imbalance in FA composition, particularly a reduction in omega-3 polyunsaturated FA and an increase of pro-inflammatory lipid metabolites [32]. Higher levels of omega-3 FA have been associated with a lower prevalence of autoantibodies in individuals at risk of RA, suggesting their protective role in the early phases of the disease [33].

Altered lipid metabolism also promotes oxidative stress and the generation of lipid peroxidation products, further amplifying immune activation. Elevated levels of reactive aldehydes derived from peroxidised polyunsaturated FA have been detected in serum, synovial fluid, and erythrocytes of RA patients, linking lipid oxidation to chronic inflammation [34]. Moreover, arachidonic acid-derived lipid mediators, including prostaglandins and leukotrienes, have been detected in inflamed joints and seem to contribute directly to synovitis, cartilage degradation and bone erosion [35].

**Fig. 3:**
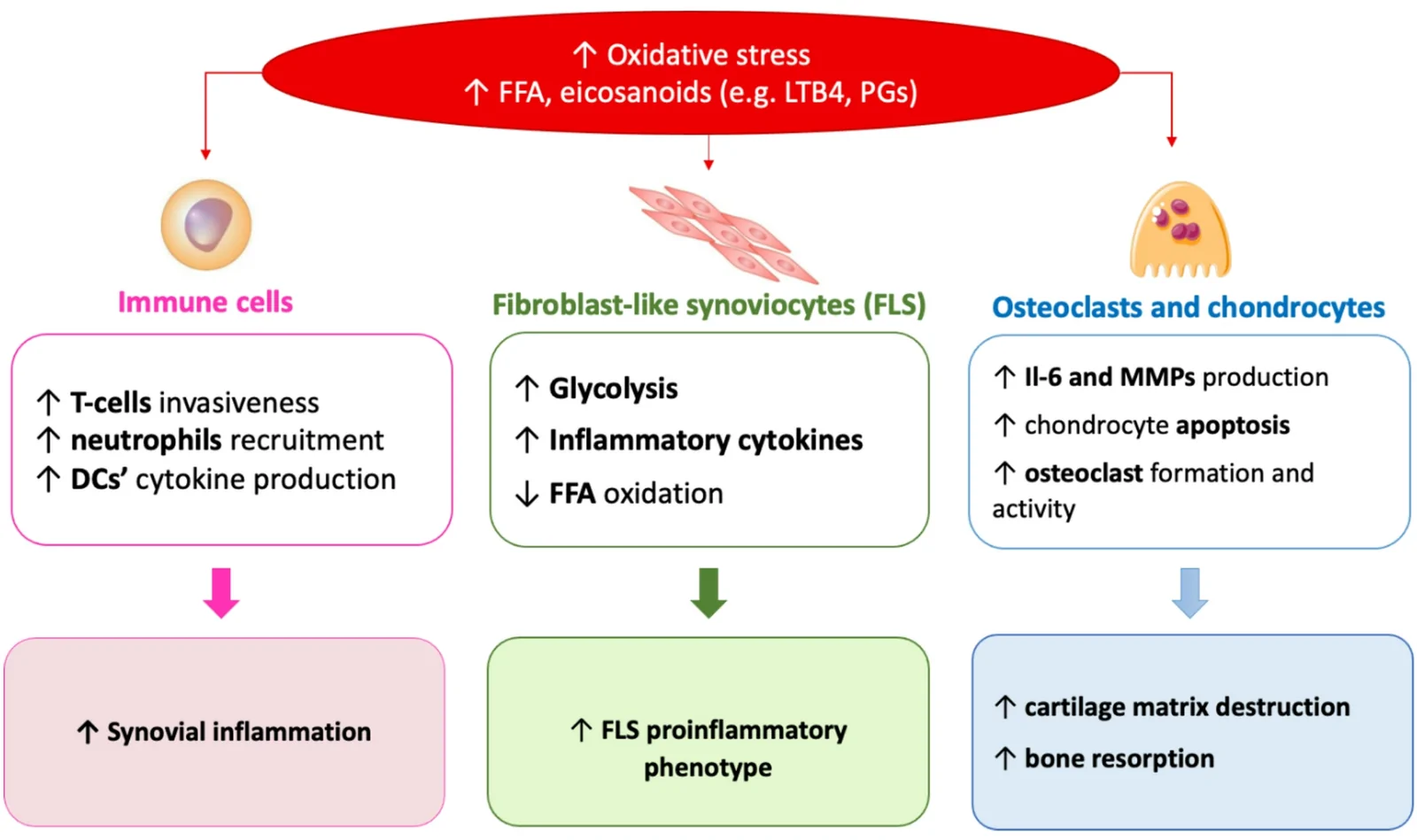
Lipid metabolism dysfunction in Rheumatoid Arthritis pathogenesis. High oxidative stress derived from chronic inflammation further leads to the production of free fatty acids and eicosanoids. These bioactive mediators can promote specific metabolic phenotypes in different cell types involved in RA pathogenesis, such as lymphocytes and fibroblast-like synoviocytes, promoting immune cell activation and recruitment and increasing synovial inflammation. Finally, increased metalloproteinase production, chondrocyte apoptosis, osteoclast activation are observed, leading to cartilage matrix destruction and bone resorption. *DC* dendridic cells, *FLS* fibroblast-like synoviocytes, *FFA* free fatty acids, *MMPs* metalloproteinases

Immune cells have shown a metabolic reprogramming that induces pro-inflammatory differentiation and persistence in the synovium. First, T lymphocytes display impaired mitochondrial respiration and increased FA synthesis, enhancing migratory capacity and tissue invasiveness [36]. Fibroblast-like synoviocytes (FLS) showed impaired mitochondrial function and altered FA metabolism, promoting an aggressive, tissue-invasive phenotype [37]. In resident joint cells, lipid mediators also promote chondrocyte apoptosis and extracellular matrix degradation, enhance osteoclast differentiation, and inhibit osteoblast function, contributing to bone resorption [35, 38] (Fig. 3). These findings demonstrate that lipid metabolic dysfunction is tightly integrated into RA pathogenesis, representing both a consequence and an active driver of chronic inflammation and articular damage, supporting the use of metabolic pathways as an adjunctive strategy to modulate disease activity in RA.

## Impact of RA therapies on lipid metabolism

The reduction of systemic inflammation represents one of the main targets in CV risk reduction in RA patients. However, antirheumatic therapies show heterogeneous effects on lipid metabolism, underlining the need to carefully interpret lipid changes observed during treatment.

GC remain widely used for disease control, particularly in the acute phases, but their metabolic effects on lipids are mainly unfavourable. First, they enhance hepatic lipogenesis and impair lipid clearance, increasing circulating levels of TG and LDL cholesterol; on the other hand, prolonged GC treatment contributes to endothelial dysfunction and oxidative stress, further amplifying atherosclerotic risk [39]. These effects reinforce the need to limit cumulative steroid exposure whenever possible.

Among csDMARDs, methotrexate displays the most favourable cardiometabolic profiles. It consistently correlates with reduced CV morbidity and mortality, likely mediated by control of inflammation and improved lipoprotein handling rather than by direct lipid lowering. Importantly, methotrexate appears to enhance macrophage cholesterol efflux, supporting its anti-atherogenic potential despite minimal changes in standard lipid measurements [40, 41].

Biologic and targeted synthetic DMARDs profoundly influence lipid metabolism, largely by reversing inflammation-driven hypocholesterolaemia. Particularly, TNF inhibitors and IL-6 receptor antagonists are more strongly associated with increases in total and LDL cholesterol levels [42].

A large cohort study showed a reduced incidence of CV events among RA patients receiving TNF inhibitors compared with biologic-naïve patients [43]. Moreover, a recent meta-analysis reported no difference in the incidence of CV events in patients treated with abatacept compared to TNF inhibitors, while a significantly reduced risk was observed with IL-6 inhibitors, suggesting a general cardioprotective effect despite lipid elevations [44].

IL-1 inhibitors, such as anakinra and canakinumab, are rarely used in RA. However, the role of IL-1 in atherosclerosis has been largely studied, since IL-1 blockade reduces endothelial activation, leukocyte recruitment and downregulates the IL-6 signalling. The CANTOS trial demonstrated a reduction in recurrent CV events with IL-1β inhibition among patients with prior myocardial infarction, supporting the validity of targeting inflammation for CV risk reduction [45].

Rituximab, an anti-CD20 monoclonal antibody indicated in severe RA, appear metabolically neutral, with no relevant adverse effects on lipid levels and CV risk.

Finally, the influence of JAK inhibitors on CV risk remains to be further understood. It has been observed that JAK inhibitors initially increase lipid levels, particularly LDL and HDL, while generally maintaining a stable LDL/HDL ratio [46]. These changes occur mainly in the early phase after treatment initiation and tend to stabilise over time. From a pathogenetic perspective, the anti-inflammatory effect of JAK inhibitors could reverse the lipid paradox, thereby increasing cholesterol levels.

Beyond lipid levels, JAK inhibitors have been associated with a higher incidence of CV and thromboembolic events compared to TNF inhibitors. Data from real-world evidence linked the overall increased risk to the presence of baseline CV risk factors [47]. The European Medicines Agency has therefore recommended careful patient selection and risk stratification, limiting their prescription in patients aged 65 years or more and in the presence of other CV risk factors.

In this context, a recent analysis proposed that suboptimal management of LDL‑cholesterol may contribute to part of the safety signal observed with JAK inhibitors [48]. This hypothesis highlights the potential importance of proactive lipid monitoring and optimization of lipid‑lowering therapy in selected patients receiving JAK inhibitors.

Overall, lipid alterations observed during RA treatment should not be viewed as isolated adverse effects but rather as part of an interaction between inflammation, immune modulation, and metabolic regulation.

## Strategies for cardiometabolic risk assessment and monitoring in RA

In patients with RA, cardiometabolic risk assessment requires a structured and dynamic approach that accounts for traditional CV risk factors, disease activity, inflammatory burden, and treatment-related metabolic effects. From a clinical practice perspective, routine metabolic screening should be integrated into rheumatologic care and adapted over time.

Currently, specific monitoring strategies from the European League Against Rheumatism (EULAR) and the American College of Rheumatology (ACR) for glucose monitoring in patients with rheumatic diseases are limited and largely follow those applied to the general population. Standard screening tests include fasting FBG, an oral glucose tolerance test (OGTT), and HbA1c determination and the current consensus recommends screening for T2DM in individuals with known risk factors [49]. This may be particularly important in pregnant patients with inflammatory arthritis, where early screening for gestational diabetes is mandatory to reduce maternal and foetal risks [50].

Screening for prediabetes and T2DM should be initiated at age 25. However, in adults with a BMI indicating overweight or obesity, screening is recommended regardless of age, if at least one other risk factor is present [49].

As previously discussed, the RA lipid profile shows a paradoxical, non-linear correlation with inflammation, especially in active disease. Therefore, its monitoring needs to be contextualized in the clinical setting.

EULAR guidelines advocate assessing total cholesterol (TC) and HDL to stratify CV risk once the patient has reached a stable disease state. Evidence suggests that the TC/HDL ratio is a more sensitive predictor of CV disease in RA than lipid components evaluated separately [51].

Finally, lipid profile monitoring needs to be implemented according to ongoing treatments.

The Systematic Coronary Risk Estimation 2 (SCORE2) system has been developed to estimate 10-year CV risk in the general population. However, this tool in RA carries a high risk of underestimation since it does not account for specific disease-related risk factors. EULAR recommendations suggested a 1.5 multiplication factor for RA patients to improve risk prediction [51], though the risk of underestimation remains considerable.

A more specific risk stratification has been proposed by the working group of cardiovascular pharmacotherapies of the European Society of Cardiology (ESC). They stratified RA patients into Low-risk (LR-RA) and High-risk (HR-RA) groups based on disease activity, disease-related negative prognostic factors, and cumulative steroid treatment [52]. Finally, both EULAR and ESC recommend the integrated use of carotid ultrasound to detect subclinical atherosclerosis.

EULAR recommends a CV risk assessment at least once every five years, with reassessment following any significant changes in antirheumatic therapy, while the Italian CORDIS group proposed a new algorithm based on a disease-specific CV risk stratification. Patients are stratified in low, medium and high-risk, accounting for disease activity, disease-related prognostic factors, ongoing antirheumatic treatments and presence of traditional CV risk factors. While low-risk patients follow the EULAR guidelines, CV risk in medium and high-risk patients should be assessed at the time of the visit. Carotid ultrasound is always recommended for medium-risk patients, while a cardiologic referral is mandatory for high-risk patients [53].

Given the lack of a defined timing for integrating inflammation, antirheumatic therapies, and metabolic monitoring in RA, we recognize that cardiometabolic management must be implemented through the coordination of a clearly defined multidisciplinary panel, as recently outlined [3]. Therefore, we propose an algorithm derived from a synthesis of current recommendations and scientific literature examined in this review, reporting the highest level of evidence available (Table 1). We defined different clinical settings commonly encountered not only by rheumatologists but also by other specialists, such as cardiologists, diabetologists, and internists, when managing patients with RA and multiple comorbidities.

**Table 1:** Proposed algorithm for metabolic and CV risk monitoring in RA

Clinical Setting	Suggested Assessment	Timing	Evidence
Baseline RA Evaluation	FBG, HbA1c, TC, HDL, TG, LDL, Lp(a)*	At diagnosis or prior to treatment escalation	Guidelines/recommendations (EULAR 2015/2016; ESC/EAS 2019; ESC 2021; ADA 2024)
High Disease Activity*	Lipid profile (TC, HDL, TG, LDL)	Re-evaluate 8–12 weeks *after* achieving low disease activity or remission*	Recommendations (EULAR 2015/2016); Observational studies
GC Therapy	FBG or capillary glucose; HbA1c	FBG: at baseline, monitoring tailored on the GC dose and individual risk; HbA1c: at baseline and 3 months post-initiation	Guidelines (ADA 2024); expert opinion [54]
	Lipid profile (TC, HDL, TG, LDL)	At baseline, after 4–12 weeks and every 3–12 months according to individual risk	Guidelines (ESC/EAS 2019)
IL-6i or JAKi Initiation	Lipid profile (TC, HDL, TG, LDL)	4–12 weeks after treatment start	RCTs; Drug labels (EMA/FDA)
CV Risk Assessment	SCORE2 (with 1.5x multiplier in active RA)	Once every 5 years or upon significant change in disease activity. At the time of the visit if RA negative prognostic factors or chronic NSAIDS/GC treatment	Recommendations (EULAR 2015/2016); CORDIS position paper (2023)
	Carotid ultrasound	Once every 5 years or at the time of the visit for medium-risk patients according to CORDIS	

*ADA* American Diabetes Association, *ApoB* Apolipoprotein B, *BMI* Body Mass Index, *CORDIS* Cardiovascular, Obesity and Rheumatic Diseases Italian Study, *EAS* European Atherosclerosis Society, *EMA* European Medical Agency, *ESC* European Society of Cardiology, *EULAR* European League Against Rheumatism, *FBG* Fasting Blood Glucose, *FDA* Food and Drug Administration, *GC* glucocorticoids, *GDM* Gestational Diabetes Mellitus, *HbA1c* Glycated Hemoglobin, *HDL* High-Density Lipoprotein, *IL-6i* Interleukin-6 inhibitors, *JAKi* Janus Kinase inhibitors, *LDL* Low-Density Lipoprotein, *Lp(a)* Lipoprotein (a), *OGTT* Oral Glucose Tolerance Test, *RA* Rheumatoid Arthritis, *SCORE2* Systematic Coronary Risk Evaluation 2, *T2DM* Type 2 Diabetes Mellitus, *TC* Total Cholesterol, *TG* Triglycerides, *CV / CVD* Cardiovascular / Cardiovascular Disease.

*Defined according to the ACR/EULAR (Studenic P et al. 2022): Boolean 2.0 remission (tender joint count ≤ 1, swollen joint count ≤, C-reactive protein ≤ 1 mg/dL, Patient Global Assessment ≤ 2 cm) or Simplified Disease Activity Index (SDAI) ≤ 3.3 or Clinical Disease Activity Index (CDAI) ≤ 2.8.

## Cardiometabolic risk management in RA

Following these recommendations, CV risk management in these patients should primarily focus on optimal disease control and minimizing systemic GC use.

Regarding pharmacological treatment, the use of antidiabetic agents in RA follows the recommendations for the general population, although their role in inflammatory diseases has yet to be fully elucidated. In fact, therapies as metformin and, more recently, sodium-glucose cotransporter-2 (SGLT2) inhibitors, have also demonstrated anti-inflammatory and cardioprotective properties [55].

Lipid-lowering therapy for primary prevention in RA should be guided by a comprehensive CV risk assessment that accounts for disease activity, inflammatory burden and traditional risk factors. According to the ESC guidelines, LR-RA patients can follow the general population guidelines, with LDL < 115 mg/dL for all individuals at low-to-moderate CV risk, whereas HR-RA patients should be classified into a one-level higher ESC category, implying stricter LDL targets [56].

### Focus on: dietary and vitamin D supplementation

An appropriate management also includes lifestyle changes, such as a healthy diet, smoking cessation, and regular physical activity. Micronutrient deficiency has been documented in inflammatory arthritis and linked to higher disease activity [57]. Beyond its well-known function in calcium metabolism, vitamin D exerts important immunomodulatory effects through the vitamin D receptors (VDRs) expressed in several immune cell populations. Results from experimental and translational studies indicate that vitamin D signalling may regulate inflammatory responses in many immune-mediated diseases, including RA, by modulating cytokine production, inhibiting pro-inflammatory pathways, and promoting immune tolerance. Different molecular pathways have been identified, including their role in T-cell maturation, leading to a transition from a pro-inflammatory to an anti-inflammatory phenotype. It also showed reduced macrophage and dendritic cell activation and NF-κB inhibition, a key signalling pathway in RA pathogenesis [58]. Several other mechanisms have been proposed, including interactions between vitamin D signalling and oxidative stress, mitochondrial function, and epigenetic regulation of immune-related genes. However, whether these pathways are recognized in RA and contribute to its pathogenesis remains to be established [58]. Finally, more recent studies suggest that vitamin D reduces IR through both protective effects on pancreatic beta cells and actions on metabolically relevant tissues, including insulin-sensitive tissues such as skeletal muscle and adipose tissue [59]. In RA, dietary supplementation with omega-3 FA and vitamin D in combination with standard therapy showed improved clinical outcomes compared to pharmacological treatment alone [60] and correlated with lower arterial stiffness in RA patients, emphasizing their on both disease activity and CV risk [61].

### Focus on: glucagon-like peptide-1 receptor agonists

Glucagon-like peptide-1 receptor agonists (GLP-1 RAs) are a class of drugs used to treat T2DM and obesity. More recently, tirzepatide, an agonist of glucose-dependent insulinotropic peptide (GIP) and GLP-1 receptors, has been approved for clinical use [62]. GLP-1 is an incretin hormone secreted by intestinal endocrine cells, which starts its signalling cascade through binding to the GLP-1 receptor (GLP-1R). After binding to the GLP-1 receptor (GLP-1R), GLP-1 directly enhances glucose-dependent insulin secretion from pancreatic β-cells, thereby modulating glucose metabolism. In addition, GLP-1 promotes satiety and delays gastric emptying, leading to significant body weight reduction [63].

Beyond its metabolic effects, available data suggest that GLP-1 plays a role in regulating systemic inflammation and immune regulation. GLP-1R is expressed in several immune cell types, including macrophages, T and B lymphocytes. GLP-1RA has been shown to reduce the production of pro-inflammatory cytokines, such as TNFα, IL-1β, and IL-6, both in vitro and in vivo models [64]. GLP-1R has also been identified in chondrocytes, and its activation resulted in suppressing inflammatory pathways involved in osteoarthritis, including the NFk-B and the production of IL-6 and TNFα. Based on this evidence, GLP-1 RAs are under evaluation as potential disease-modifying drugs in autoimmune diseases [65].

Whether GLP-1 RAs reduce RA disease activity through immune modulation, metabolic improvement, and weight loss, or both remains to be defined. Preclinical studies demonstrate anti-inflammatory and cytoprotective effects of GLP-1RA on FLS stimulated with pro-inflammatory cytokines (Table 2) [66–68]. However, well-designed randomized controlled trials are needed to confirm their therapeutic role in RA.

**Table 2:** Summary of basic science studies investigating the effects of GLP-1 receptor agonists in rheumatic diseases

Study	Drug	Target	Results
Du X et al., Int Immunopharmacol 2019	Lixisenatide	FLS stimulated with IL-1β	$\:\:\downarrow\:$ Oxidative stress, mitochondrial dysfunction, apoptosis $\:\:\downarrow\:$ Pro-inflammatory cytokines (TNFα, IL-6, IL-8) Inhibition MMPs Suppression of key pro-inflammatory pathways (JNK, AP-1, NF-κB)
Tao Y et al., IUBMB Life 2019	Exenatide	FLS stimulated with TNFα	$\:\downarrow\:$ Oxidative stress, mitochondrial dysfunction $\:\downarrow\:$ Pro-inflammatory cytokines (IL-1β, IL-6, MCP-1, HMGB-1) Inhibition MMPs Inhibition of key pro-inflammatory pathways (p38/MAPK and IκB-α / NF-κB)
Zheng W et al., Int Immunopharmacol 2019	Dulaglutide	FLS stimulated with TNFα	$\:\downarrow\:$ Oxidative stress, mitochondrial dysfunction $\:\downarrow\:$ Pro-inflammatory cytokines (IL-1β, IL-6, MCP-1, HMGB-1) Inhibition MMPs Inhibition of key pro-inflammatory pathways (JNK/ NF-κB)

Basic science studies demonstrate that GLP-1 receptor agonists, including lixisenatide, exenatide, and dulaglutide, reduce oxidative stress, mitochondrial dysfunction, and pro-inflammatory cytokine production in fibroblast-like synoviocytes (FLS) stimulated with pro-inflammatory mediators, while inhibiting key inflammatory signaling pathways (JNK, AP-1, NF-κB, p38/MAPK).

## Conclusion

RA represents a model of interplay between metabolic dysfunction and inflammation, in which glucose and lipid abnormalities must be interpreted in the context of underlying disease mechanisms.

Clinicians have to be aware that chronic systemic inflammation profoundly reshapes cardiometabolic risk, frequently masking high-risk profiles. Alterations in glucose and lipid metabolism should be considered part of RA-associated metabolic disease rather than incidental comorbidities.

This has clear implications for cardiometabolic screening strategies, which in these subjects should incorporate disease activity, inflammatory burden, and treatment exposure.

From a metabolic perspective, RA also challenges the validity of traditional CV risk calculators, which may substantially underestimate risk in the presence of active inflammation and qualitative lipoprotein dysfunction. Moreover, anti-inflammatory therapies commonly used in RA exert heterogeneous effects on glucose and lipid metabolism. Therefore, their values should be interpreted in the context of disease activity, inflammatory burden, lipoprotein function, and ongoing treatments.

In conclusion, incorporating inflammatory status into metabolic assessment represents a critical step toward more accurate CV risk stratification and personalized prevention in RA. These considerations also support the need for a multidisciplinary approach to cardiometabolic risk management in RA, involving rheumatologists, endocrinologists, cardiologists, internal medicine, and primary care.
